# Intrathoracic rib: rare rib anomaly, review of the literature and proposal for classification

**DOI:** 10.7150/ijms.63828

**Published:** 2021-10-16

**Authors:** Xuhong Xue, Sheng Zhao, Kai Li, Bin Zhao

**Affiliations:** 1 Department of Orthopedics, The Second Hospital of Shanxi Medical University, Taiyuan, Shanxi, 030001, P.R. China.; 2 Department of Orthopedics, The Second Hospital of Shanxi Medical University, Taiyuan, Shanxi, 030001, P.R. China.

**Keywords:** ribs, congenital abnormalities, review, literature, classification

## Abstract

**Background:** Intrathoracic ribs are very rare congenital anomalies, and often discovered incidentally on chest X-ray. Since its first description by Lutz in 1947, approximately 50 cases have been reported in the literature till date. The aim is to review the all reported intrathoracic ribs, summarize their clinical features, and propose a potential classification.

**Methods:** All relevant literatures were searched and reviewed. The terms include intrathoracic rib, intrathoracic bifid rib, trans-thoracic rib and intrathoracic rib anomaly. We have summarized the first finding events, origination, distribution, related anomalies and imaging features of intrathoracic rib, and propose an updated classification.

**Results:** The patients' age at initial finding was from six weeks to 79 years old. Of all, sixty percent was less than 30 years old. There was no difference in gender. Most of them were reported by authors in western countries (85.3%, 58/68), and incidental findings by radiologist and respiratory physician. The intrathoracic rib occurs more frequently on the right side, and is usually single and unilateral. According to the new classification, type I and II was account for 45.6% and 35.3%, respectively.

**Conclusion:** Intrathoracic rib is rare findings in clinical practice. It is useful that radiologists or clinician are familiarized with the imaging appearances of these malformations. These anomalies reflect some disturbances during the embryo development, leading us to propose a potential classification that could contribute to a better understanding of this rib anomaly.

## Background

Rib anomalies are reported to account for 1% of the population 1. However, intrathoracic rib identified is very rare. It is an unusual thoracic cage anomaly manifested a normonumerary or supernumerary rib or a bifid rib, which usually follows an abnormal course in the thoracic cavity 2. Since its first description by Lutz in 1947 3, only approximately 50 cases have been reported till date 4. In most of all cases, they are asymptomatic, but they may be associated with developmental abnormalities of ribs and vertebrae.

There is a wide spectrum of morphologic variants of rib anomalies. Unfortunately, the boundary between malformation and anatomical variation remains unclear, resulting in misdiagnosis 5. Furthermore, these rib anomalies are reported in the publication mainstream as case report, typical imaging or brief literature review. To the best of our knowledge, there are few studies to analyze a larger number of cases and morphologic features, to determine their prevalence, distribution or association with other malformations. More than a decade ago, Kamano H et al described a classification of intrathoracic rib based on embryologic origination 6. However, it was incomplete and several types of intrathoracic rib have not been included.

We aimed to review all reported literatures of intrathoracic rib, in order to summarize their clinical features and to raise awareness on the rare congenital rib anomalies. Further, based on their imaging features and the existing data in developmental biology, a new classification system was proposed as an update.

## Methods

### Literature search

A systematic search was conducted in Medline, Embase, EBSCOhost, Google scholar, Web of Science and Scopus for literature published from January 1945 through December 2020. Keywords and medical subject headings related to the condition were identified prior to initiating the search. The MESH search terms for MEDLINE included: (intrathoracic rib) OR (bifid intrathoracic rib) OR (transthoracic rib) OR (intrathoracic rib anomaly). Gray literature, including books and conference papers, were collected and these studies were included if they met inclusion criteria. No linguistic restriction was imposed on the search. The unpublished investigations were not included.

### Inclusion and exclusion criteria

We reviewed published studies according to the following criteria: (1) Intrathoracic rib is defined as a rib anomaly in which a normal, supernumerary, or a bifid rib lies within the chest cavity; (2) subjects who had no age restriction at initial presentation; (3) the study reported at least one case with intrathoracic rib; (4) The types of treatment included surgical and non-surgical interventions, Excluded criteria were as following: (1) Editorials and comments were excluded. (2) Patients with rib deformity lies out the chest cavity were excluded.

**Figure 1:**
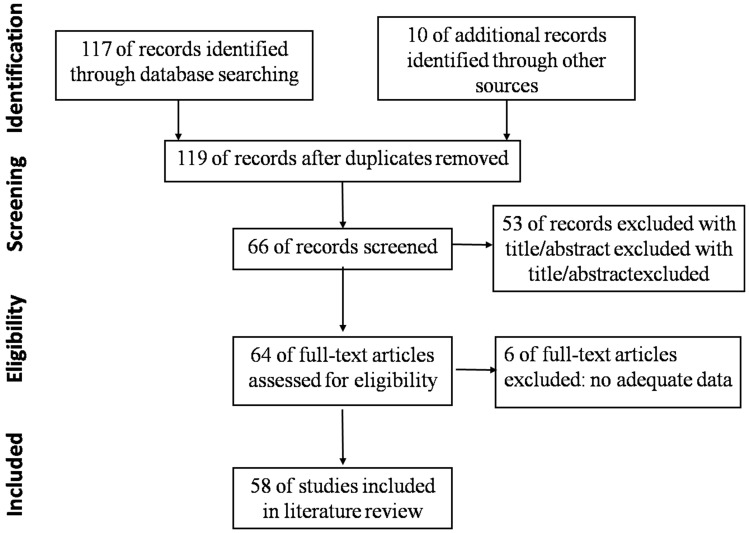
Flow chart of identifying and including studies.

The flow chart shows the study selection process (Fig. 1). Initially, 119 articles were included by search strategy. After reviewing titles and abstracts, sixty-six articles remained for screening based on the inclusion criteria. Of these sixty-six articles, sixty-four full text articles were selected for further evaluation. Six studies were excluded after reviewing full-texts. Finally, fifty-eight eligible studies were identified, relevant to this topic 2-4, 6-60. 5 papers were in German, 1 in Polish, 2 in French, 1 in Portuguese, 1 in Spanish, 1 in Japanese, and other 47 papers were in English.

Demographic and radiographic data, including age, gender, sites of rib anomalies, as well as other related abnormalities, were record. As they were diagnosed, initial finding events and related symptoms were of great importance and also record, including chest pain, fever, cough, hemoptysis, dyspnea, etc. Other than that, national and department distribution of authors in these literatures was analyzed. Based on the imaging features of rib anomalies and existing data in developmental biology, a new classification of anomalies was proposed.

## Results

### General information

**Figure 2:**
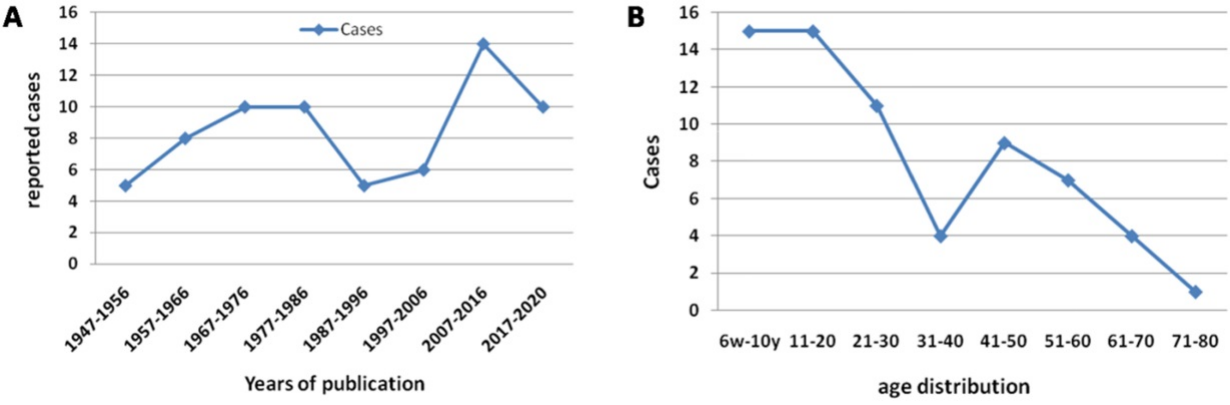
The tendency of growth cases per ten years (**A**). The age distribution of intrathoracic rib (**B**).

The details of demographic and radiographic data were summarized (supplemental materials). In all, sixty-eight cases were described; thirty-one (45.6%, 31/68) occurred in males and thirty-six (52.9%, 36/68) in females, one case (1.5%, 1/68) was no detail gender given. Only one article included 3 cases; 7 articles included 2 cases; and other 50 papers reported single case. Since its first report in 1947, average nine cases (13.2%, 9/68) were reported per decade. The patients' age at initial finding was from six weeks to 79 years old (Figure 2). Of all, sixty percent was less than 30 years old. There was no difference in gender. Eight-five percent were reported by authors in western countries, and incidental findings by radiologist and respiratory physician.

### First finding events and related symptoms

In most cases, these anomalies are incidental findings and asymptomatic. However, chest pain, breathing difficulty, and hemoptysis have been reported in patients with intrathoracic ribs. Fifty-two cases (76.5%, 52/68) had description of first finding event. More than one third of patients had incidental finding due to chest X-ray for respiratory symptom, including fever, cough, hemoptysis, dyspnea, tuberculosis, etc. Nine cases (13.2%, 9/68) were found due to chest pain, 8 cases (11.8%, 8/68)were found in routine health check-up, 9 (13.2%, 9/68) were found in preoperative or on admission routine checkup, 4 (5.9%, 4/68) were following-up for other disease, and 1(1.5%, 1/68) was swallowed a cent piece.

In most of cases, it seems probable that these symptoms were unrelated to the intrathoracic rib. Relevant symptoms were present in only two cases (2.9%, 2/68). In Marbut's case, a 20-year-old female had pain in the region of the scapula on the side of the anomaly. However, she presented the symptoms from an injury to the thoracic spine 8. So it was hard to say whether related to rib anomaly. Another case was reported by Coyan G in 2016, which was a supernumerary intrathoracic rib causing increasing chest pain. The patient underwent robotic-assisted video-assisted thoracoscopic resection and preoperative symptoms were completely resolved upon discharge 51.

### Location of rib anomalies

The intrathoracic rib occurs more frequently on the right side, and is usually single and unilateral. Forty cases (58.8%, 40/68) were right-sided and twenty-seven (39.7%, 27/68) on the left; the ratio was 3:2. Only one case had bilateral intrathoracic ribs (1.5%, 1/68). The location of intrathoracic rib was summarized in Tables 1and 2. In most of cases, it arises between the 2^nd^ and 8^th^ thoracic vertebra; or between first and seventh proximal or distal rib. There were two cases no articulation with rib or vertebra, named floating intrathoracic rib. One was found incidentally and asymptomatic; a 44-year-old male with a pericardiac rib (along the left ventricular wall) and without any osseous union with a vertebra or rib 45. Another one was reported by Peterson MS in 1993, which was extended from the level of left sixth posterior interspace to the level of the posterior aspect of ninth rib without osseous attachment 34.

There was only one case of a bilateral anomaly, which had four thoracic cavity-like compartments separated by two intrathoracic rib extending inferolaterally in bilateral hemithorax. Reconstruction CT showed there was a bifurcated rib articulated with anterior aspect of the T5 vertebra, extending toward on anterior inferolaterally and protruding into the thorax cavity 58. Two cases had two anomalous intrathoracic ribs on the same side, one articulating with T6 and the other with T7 vertebra 9. Another case has two left-sided intrathoracic ribs, one arising from posterior segment of the 7^th^ rib and other from 5^th^ rib 28. Only one case with four intrathoracic ribs from most dorsal part of the normal rib was found during autopsy by anatomist in 2013 50. Two cases presented rib protruded into the thorax cavity and resulted in lung collapse 9, 47.

### Imaging features and other related anomalies

**Figure 3:**
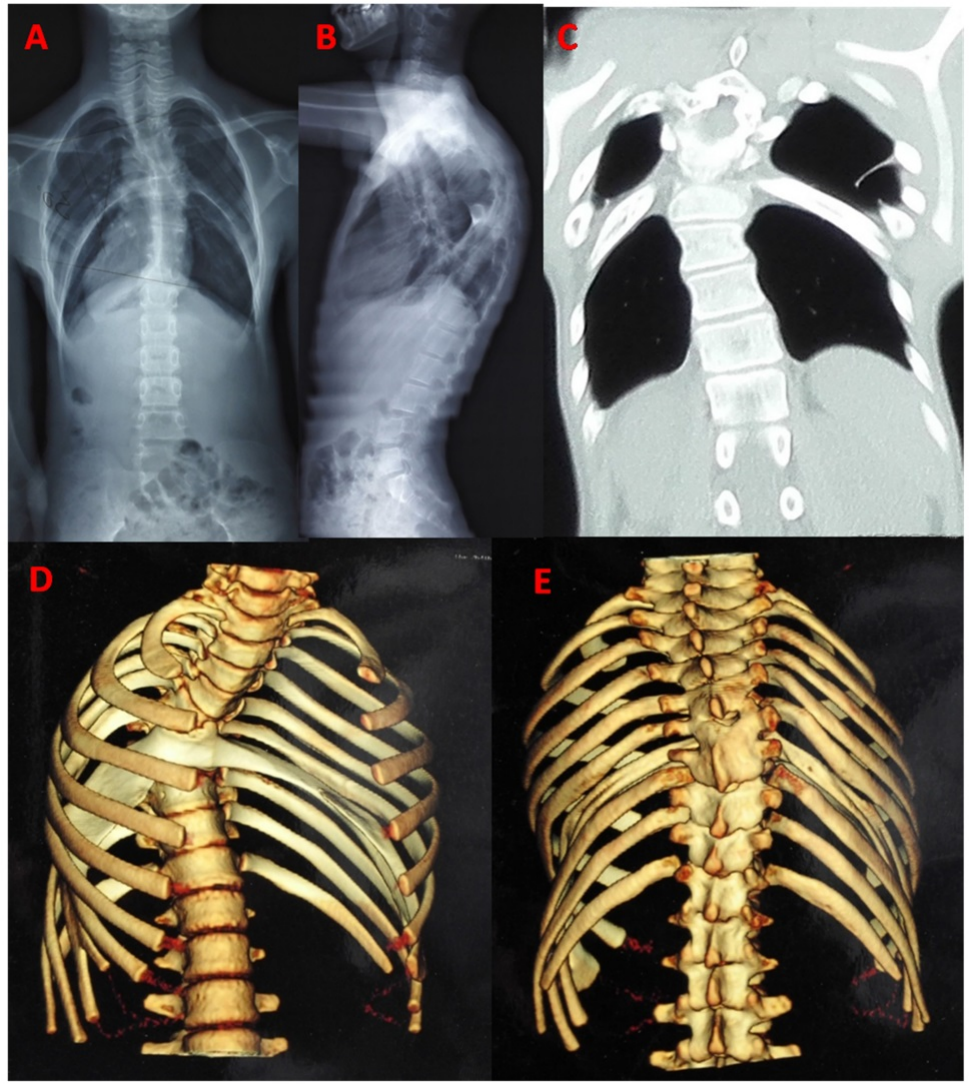
Typical image: a 13-year-old man with bilateral bifid intrathoracic rib. postero-anterior and lateral radiograph showed kyphoscoliosis in thoracic spine, and four thoracic cavity compartments were separated by bilateral intrathoracic rib (**A, B**). The coronal CT showed two deformed rib extending inferolaterally in bilateral hemithorax, forming focal additional compartment with fiber band-like attachment to pleura (**C**). CT scan with 3D reconstruction showed bifurcated rib articulated with anterior aspect of the T5 vertebra. Distal anomalous rib had osseous connection with adjacent fused rib, extending bifid rib locally depressed into the thoracic cavity (**D, E**).

In all cases, the majority were demonstrated by plain film, conventional tomography or three dimensional CT. Three cases were confirmed by surgery, and other three were found by autopsy. In the past, three patients have been subjected to unnecessary thoracotomy. Expansion of the rib head, tent-like pleural extension and intrathoracic fat were identified to special imaging features in previous studies 33,57 (Fig. 3). These features were very helpful for correct diagnosis of intrathoracic rib on the era of only X-ray. But now it is more easily using CT scan and three dimensional reconstructions.

On some occasions, intrathoracic ribs exhibit diaphragmatic attachments, usually in the form of a fibrotic band or fat layer. In all cases, 18 (26.5%, 18/68) were attached to diaphragm and 50 were no diaphragmatic attachment. Four of the cases (5.9%, 4/68) had fiber band attachment to the pleura with tent-like pleural extension. Only one case had fibrous band attaching to the T9 right side with thoracic scoliosis 43. Six cases (8.8%, 6/68) had mild thoracic scoliosis and two with bloc vertebra. One case had congenital thoracic kyphoscoliosis 58. Only one case had reported incidental associated findings include right-sided aortic arch 8 and liver eventration 41. Two cases had an additional rib anomaly, bilateral hypoplasia 1^st^ rib and cervical rib 9,22.

### Classification of intrathoracic rib

**Figure 4:**
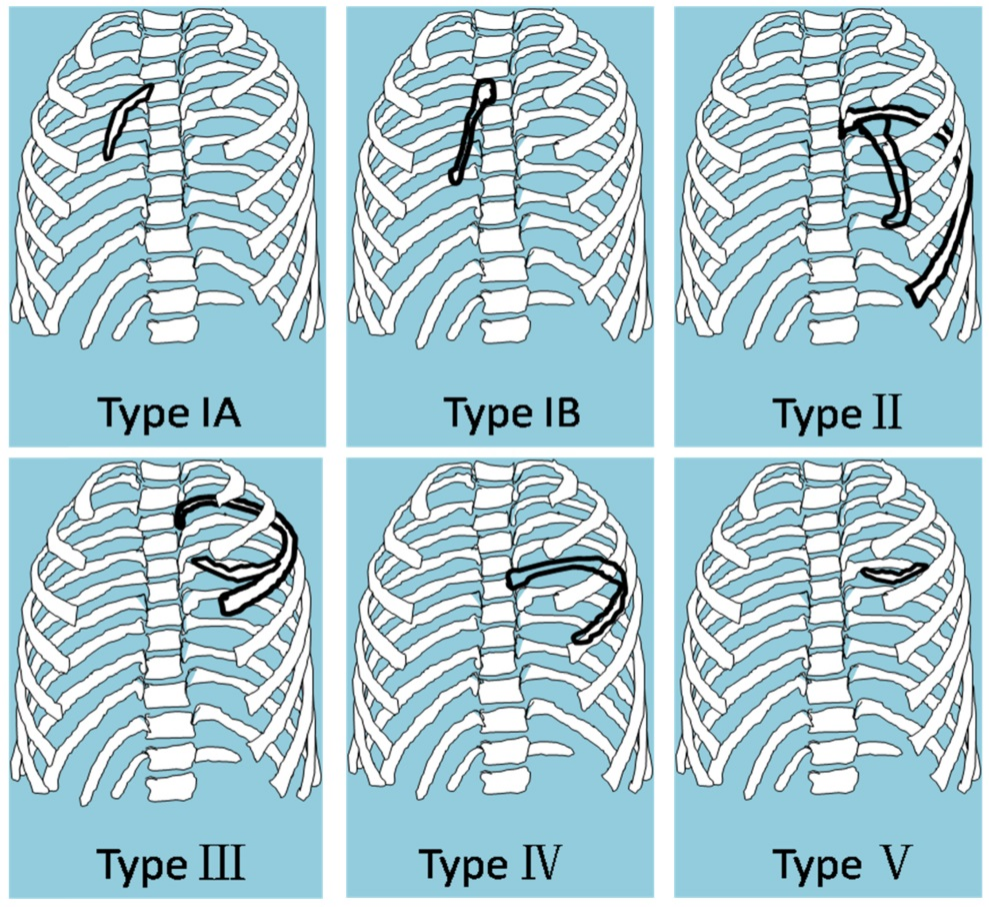
The classification of intrathoracic rib. **A**. Type I: supernumerary intrathoracic rib articulating with a vertebral body. Arising from lateral vertebral body (left or/and right). **B.** Arises from anterior vertebral body (unilateral or bilateral); Type II: supernumerary intrathoracic rib arising from proximal rib. Type II: bifid intrathoracic rib, with the bifurcation in a distal rib. Type IV: rib locally depressed into the thoracic cavity. Type V: floating intrathoracic rib, lack of osseous attachment to another rib or to a vertebra.

Based on the features of imaging, we classified the intrathoracic rib into five types. Type I: intrathoracic rib articulating with a vertebral body, including two subtypes: A. arising from lateral vertebral body (left or/and right); B. arises from anterior vertebral body (unilateral or bilateral); Type II: intrathoracic rib arising from proximal rib. Type III: intrathoracic rib arising from distal rib (bifid or forked rib). Type IV: rib locally depressed into the thoracic cavity. Type V: floating intrathoracic rib, lack of osseous attachment to another rib or to a vertebra (Fig. 4A, B).

**Figure 5:**
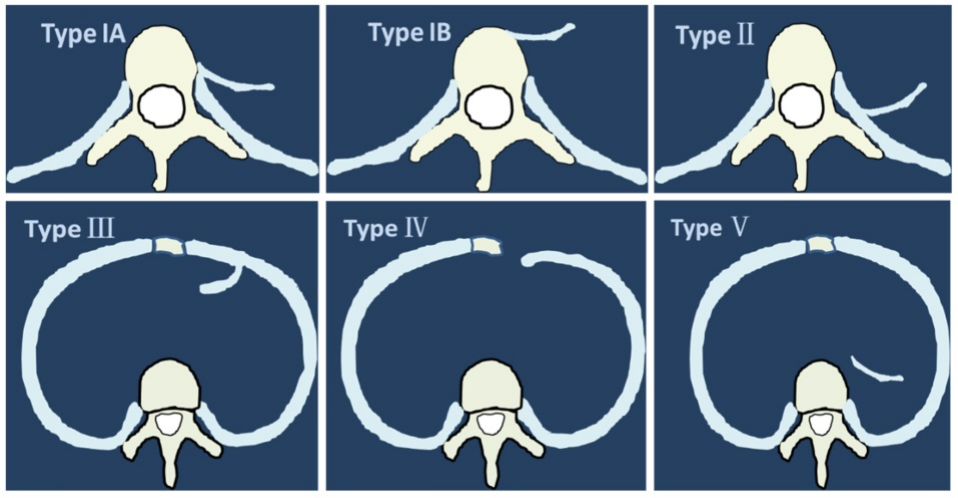
Origination of intrathoracic rib in axial view: Type IA. lateral vertebral body; Type IB. anterior vertebral body; Type II: proximal rib; Type III: distal rib. Type IV: rib locally depressed into the thoracic cavity. Type V: floating intrathoracic rib.

Of the all reported cases, we classified 24 as type IA, 4 as type IB, 26 as type II, 6 as type III, 1 as type IV, 2 as a type V (Table 3). Furthermore, there are some mixed type cases, including 1 as a type IA+type II that has two intrathoracic ribs 29, 2 as type III+IV with characteristics of a type III bifid intrathoracic rib and a type IV focal depressed intrathoracic rib 6,33, 1 as a type IA+III+IV that has two intrathoracic rib, one articulation with vertebra body and other with bifid and depressed rib 49. One is type IB+III+IV with characteristics of a type IB arising from anterior vertebral body, type III distal bifid intrathoracic rib and a type IV locally depressed intrathoracic rib. The rare case was reported by our team recently in Thorax 58. It revealed that four thoracic cavity-like compartments were separated by bifid rib extending inferolaterally in bilateral hemithorax, with fibrous band attaching to hemi-diaphragm. The patient had undergone spinal fusion surgery due to congenital kyphosoliosis. There was no special intervention for intrathoracic rib because of his asymptomatic (Fig. 5).

## Discussion

Intrathoracic rib is a very rare anomaly in which a normal, supernumerary, or a bifid rib lies within the chest cavity. The classification of intrathoracic rib has been described in previous literatures. In 1991, Hawass NE, et al first time have described intrathoracic rib anomalies as three forms: 1. Local depression of one or more ribs into the thoracic cavity. 2. Supernumerary intrathoracic rib arising from the vertebral body. 3. Supernumerary intrathoracic rib as a rare variant of rib forking 33. In 2006, Kamano H et al. suggested classification of intrathoracic rib into three categories. Type I: supernumerary intrathoracic rib articulating with a vertebral body or proximal rib. Type II: bifid intrathoracic rib. Type III: rib locally depressed into the thoracic cavity 6. However, we found these classifications were incomplete; some rare intrathoracic rib had been not included. On the other side, anomalous rib arising from anterior and lateral aspect of vertebra was completely different. We proposed the new classification of intrathoracic rib based on imaging features and embryologic disorder.

The bone structure of the ribs and vertebrae is formed in close association during gestation weeks 2 to 6 59. Ribs originate in the somites, which will give rise to specific structures for adapting to their function. This is reflected in the distinct morphology of thoracic vertebrae and in the formation of costo-vertebral joints 2. Aoyama et al defined three compartments for each rib that developed under different controls: proximal rib depending on ventral-axial tissues, vertebro-distal rib depending on the surface ectoderm, and sterno-distal rib depending on the lateral plate mesoderm. Thus the proximal part was dependent on the notochord and ventral neural tube; while the distal rib depended on the surface ectoderm. Sterno-distal rib may be partly dermomyotome. The normal development of the ribs is dependent on signaling from the dermomyotome or its derivative, the myotome 62.

During embryonic development, any abnormal signal that disturbs the interaction is believed to cause rib anomalies. In the process of rib formation, the cartilage accumulations tend to form some type of articulation with the adjacent vertebral bodies. Distribution disorders of paravertebral cartilage masses can result in abnormal ribs extend in late embryonic 63. Therefore, abnormal patterning such as fused rib, widened rib, forked rib, asymmetrically positioned ribs, and fewer or absent ribs were formed. It is not difficult to understand that the radiographical classification from above mentioned different embryological origins of patterning of intrathoracic rib. It is suggested that intrathoracic rib results from incomplete fusion of the cranial and the rostral segments of the sclerotome during embryological development.

From the viewpoint of genetic and molecular biology, the control of rib development appears to be regulated through Myf5/Myf6 expression 64. Referring to the classification of intrathoracic rib we propose, type I is associated with vertebrae abnormality; Hox gene family is reported to be important in the development of vertebra 65. TypeⅡ is arising from proximal rib. It was reported that Pax-1 is responsible for proximal rib development 62. The type III condition reflects a bifid rib originating from a distal rib. Aoyama et al. thought there was some evidence that the Myf5 knockout mouse lacked distal ribs. Pax-3-deficient mice also exhibit severe distal rib defects 63. Type IV is the depressed intrathoracic rib, which is believed to arise from a combination of alterations in gene expression and a pressure effect applied to the chest wall 33. Type V is floating intrathoracic rib. We postulate improper distribution of cartilage masses could lead to no articulation with corresponding vertebra body. The specific developmental biological mechanism needs further investigation.

With the development of imaging technology, use of CT scan imaging can identify the extrapleural location, the origin and path of the intrathoracic rib. It also further characterizes the potential associated findings such as intrathoracic fat or the presence of a fibrous diaphragmatic attachment. CT reconstructions allow a better appreciation of the curving tubular-like nature of the intrathoracic rib and its similarity to other ribs.

## Conclusion

Intrathoracic rib is rare findings in clinical practice. It is useful that radiologists or clinician are familiarized with the imaging appearances of these malformations. The first finding events, origination, distribution, related anomalies and imaging features of intrathoracic rib were analyzed comprehensive for all reported cases. Furthermore, these anomalies reflect some disturbances during the embryo development, leading us to propose a potential classification that could contribute to a better understanding of this rib anomaly.

## Supplementary Material

Supplementary table S1.

## Figures and Tables

**Table 1:** The rib origin of intrathoracic rib

	Rib							
Origin	1^st^	2^nd^	2^nd^, 3^rd^	3^rd^	4^th^	5^th^	6^th^	7^th^
Cases	2	4	1	9	10	10	4	3

**Table 2:** The vertebral origin of intrathoracic rib

	Vertebra body										
Origin	C7, T6-7	T2-3	T3	T3-4	T4	T4-5	T5	T6	T6-7	T7	T8
Cases	1	1	2	2	2	1	7	3	1	2	1

**Table 3:** Distribution of intrathoracic rib cases in proposal classification

Type	Description of morphology	NO. of Cases
I	Intrathoracic rib articulating with a vertebral body.	28
	A: arising from lateral vertebral body (left or/and right)	24
	B: arises from anterior vertebral body (unilateral or bilateral)	4
II	Intrathoracic rib arising from proximal rib	26
III	Bifid or forked Intrathoracic rib, with the bifurcation in a distal rib	6
IV	Rib locally depressed into the thoracic cavity	1
V	Floating intrathoracic rib, lack of osseous attachment to another rib or to a vertebra	2

## Abbreviations

CT: computed tomography
T: thoracic
