# Supplementary material for: Intrathoracic rib: rare rib anomaly, review of the literature and proposal for classification
Source: Int J Med Sci. 2021 Oct 16;18(16):3800–7. doi: 10.7150/ijms.63828 (PMC8579300; doi:10.7150/ijms.63828)
Supplement: Supplementary file 1 — Supplementary table S1. [file ijmsv18p3800s1.pdf]

Table 1. Summary of data on reported cases of intrathoracic rib

Author	publication year	Age	Sex	Department	Country	Side	Type	Originating	First presentation	Attached to diaphragm	Imaging	Comments
Lutz P[3]	1947	25	F		Germany	R	Ⅱ	forked 4th proximal rib	right-sided chest pain	Y		autopsy
Jacobs S[7]	1949	43	M	Medicine	USA	R	Ⅰ A	arising from T3 dorsal vertebra	bilateral far lung TB	N	X-ray	necropsy
Marbut WM[8]	1953	20	F		USA	R	Ⅰ B	arising from T6-7 anterior margins of vertebra	Chest pain in right scapular region	N	X-ray	**Relevant symptoms**
Merendino KA[9]	1956	17	F	Surgery, thoracic service center	USA	R	Ⅰ A	articulation on T6,7 ventral and slightly caudal to normal rib articulation	asymptomatic atelectasis of right lower lung	Y	X-ray	Cervical rib; thoracotomy
	1956	39	M	thoracic service center	USA	L	Ⅲ	from anterior surface of 2nd rib to dome of diaphragm	symptomatic of mitral stenosis	Y	X-ray	thoracotomy
PLUM GE[10]	1957	9	F	Radiology, pediatric, surgery	USA	R	Ⅰ A	right lateral margin of T6, downward posteriorly to the 9th intercostal space	"lung tumor"	N	X-ray	thoracotomy
WILK E[11]	1957	12	M		Germany	L	Ⅱ	Forked 6th rib		N	X-ray	
Whitlark FL[12]	1962	14	F		Canada	L	Ⅱ	posterior portion of the left 5th rib	nonproductive cough of 3 weeks	N	X-ray	right-sided aortic arch
von RJ[13]	1962	8	F		French	R	Ⅱ	Forked 4th proximal rib		N	X-ray	
	1962	5	M		French	R	Ⅰ A	arising from T5		Y	X-ray	
Weinstein AS[14]	1965	23	M	Radiology	USA	R	Ⅰ A	arising from T8		Y	X-ray	
Rosenblum	1966	25	F	Radiology	USA	R	Ⅱ	6th right posterior rib		N	X-ray	

DJ[15]	1966	17	F	Radiology	USA	L	Ⅱ	5th left posterior rib		N	X-ray	Pleural attachment present
Loewe KR[16]	1967	42	F		Germany	R	Ⅲ	Forked 3rd rib		N	X-ray	T3-4 bloc vertebra
Shoop J[17]	1969	28	F	Radiology	USA	L	Ⅱ	posterior portion of the third rib and attaching to 2nd rib anteriorly	investigation of possible pulmonary tuberculosis	N	X-ray	first description tent-like pleural extension
Heller RM[18]	1970	3	M	Radiology	USA	R	Ⅰ A	arising from T4	recurrent ear ache and sore throat	N	X-ray	
Kermond AJ[19]	1971	18	F	Radiology	Australia	L	Ⅱ	left 4th proximal rib	routine check up	N	X-ray	
González de Vega N[20]	1971	64	M		Spain	R	Ⅰ A	arising from T6		N	X-ray	
Freed C[21]	1972	3	M	Radiology	South African	R	Ⅱ	postero-inferior border of the 5th right rib; extending downwards 10th rib	swallowed a cent piece	N	X-ray	
Polga JP[22]	1974	8	M	Radiology	USA	R	Ⅰ A	ribbonlike density extending from the right side T5	Pre-op routine X-ray due to left orbit rhabdomyosarcoma	N	X-ray	
	1974	8	M	Radiology	USA	R	Ⅰ A	articulated with the T7 inferolaterally toward the right hemidiaphragm	evaluated for failure to thrive	N	X-ray	
Friedrich M[23]	1975	29	F	Radiology	Germany	L	Ⅲ	forked 5th rib		Y	X-ray	
	1975	11	M	Radiology	Germany	R	Ⅰ A	arising from T6		N	X-ray	Bilateral hypoplasia 1st rib
Kelleher	1979	44	F	Radiology	USA	R	Ⅳ	anterior portion of the right 7th	thoracic deformity on	N	X-ray	mild scoliosis

J[24]								rib	a routine check			
	1979	48	F	Radiology	USA	L	Ⅱ	posterior portion of the left 3rd rib and descended posteriorly		N	X-ray	
Hilton SV[25]	1980	6 w	F	Radiology	USA	R	Ⅱ	anteromedial aspect of right 4th and 5th posterior ribs and has bony, rib-like appearance	fever of 24 hours	N	X-ray	aseptic meningitis
Farrell JT[26]	1980	18	F	Radiology	USA	R	Ⅰ A	T3 vertebral body	urinary frequency, urgency and right side flank pain	Y	X-ray	hemorrhagic cystitis, right renal ectopia
Hashimoto K[27]	1983	46	F	Internal medicine	Japan	L	Ⅰ A	Arising from T5	Abnormal chest X-ray	N	X-ray CT	
Stark P[28]	1984	40	-	Radiology	USA	L	Ⅱ	posterior part of the left 3rd rib	chest pain and hemoptysis	N	X-ray CT	squamous cell carcinoma
Wilamski E[29]	1984	15	M	Radiology	Poland	R	Ⅰ A	T3,4 anterior vertebral body; distal forked rib	chest preventive examination	N	X-ray	Thoracic scoliosis
	1984	53	M	Radiology	Poland	L	Ⅱ	posterior part of the left fifth rib	exacerbation of duodenal ulcer; spine pain	N	X-ray	Active duodenal ulcer
	1984	59	F	Radiology	Poland	L	Ⅰ A+ Ⅱ	two left intrathoracic rib: 1. posterior segment of the 7th rib; 2.from 5th rib and diagram	subarachnoid haemorrhage and severe pulmonary edema	Y	X-ray	two left-sided intrathoracic rib
Von E. Wilk [30]	1986	12	M	Radiology	Germany	L	Ⅲ	5th forked distal rib		N	X-ray	
Blery M[31]	1988	27	F	Radiology	French	R	Ⅱ	posterior part of right 2nd rib		N	X-ray CT	

Trigaux JP [32]	1990	32	F	Radiology	Belgium	R	Ⅱ	Arising from 4th and 5th rib, bifid, bony rib-like appearance	dyspnea with nonspecific retrosternal pain	N	X-ray CT	
Hawass NE [33]	1991	55	F	Radiology	Saudi Arabia	R	Ⅲ+Ⅳ	lower arm of the 1st rib forking to be intrathoracic in location	preemployment check up	N	X-ray CT	First introduce intrathoracic fat feature
Peterson MS[34]	1993	70	M	Radiology	USA	L	Ⅴ	Floating rib, no articulation with rib or vertebra	Pre-op routine chest X-ray for THA revision	Y	X-ray CT	First intrathoracic rib no articulation with rib or vertebra
Kamaruddin K[35]	1995	11	F	Radiology	UK	L	ⅠA	Arising from T5	right mild chest pain	N	X-ray	
Laufer L[36]	1999	15	M	Diagnostic Radiology	Israel	L	Ⅲ	articulated with the anterior enlarged rib segment.	routine chest examination	N	X-ray, CT+3D	Fibrous attachment to the pleura
MacEneaney P[37]	2001	58	M	Radiology	USA	L	ⅠA	articulates with T5	hemoptysis	Y	X-ray, CT	right hilar mass; upper lobe collapse
	2001	27	F	Radiology	USA	R	ⅠA	Arising from T5	shortness of breath and pleuritic chest pain	N	X-ray, CT	mild thoracic scoliosis; SLE
Barreiro T[38]	2003	23	M	Pulmonary and Critical Care Medicine	USA	R	ⅠA	Arising from T5	fall down to ED	Y	X-ray	
Onbas O[39]	2005	62	M	Radiology	Turkey	R	ⅠA	3rd, ribbonlike corticated bony structure extending inward from the rib	shortness of breath, pleuritic chest pain	N	CT+3D	Thoracic scoliosis
Kamano H[6]	2006	21	F	Health Science Center	Japan	L	Ⅲ+Ⅳ	anterior-lateral portion of a deformed 4th rib	routine health check	N	X-ray, CT	

Argyriou P[40]	2007	21	M	Radiology and Thoracic Surgery	Greece	R	Ⅰ A	articulated with the T4		Y	CT+3D	
Bottosso N[2]	2008	79	M	Medical Imaging	Belgium	R	Ⅱ	6th costovertebral articulation	Routine check-up following resection of melanoma	N	X-ray, CT+3D	
Fischer B[41]	2008	7	F	Pediatric Surgery	Germany	R	Ⅰ A	T3/4 vertebra	Cough; suspected right congenital diaphragmatic hernia	Y	X-ray,MRI	liver eventration into the right thorax; T5 BFV
Watkins TW[42]	2008	8	F	Medical Imaging	Australia	R	Ⅰ B	articulated with the anterior aspect of the T4 and T5	investigation of URTI	Y	X-ray, CT+3D	mild scoliosis
Apaydin M[43]	2009	37	M	Radiology	Turkey	R	Ⅰ B	articulation with the anterior 3rd vertebra body	routine check-up	N	X-ray, CT+3D	fibrous band attaching to the T9 right side; scoliosis
Chung JH[44]	2009	67	F	Radiology	USA	R	Ⅱ	5th proximal rob	routine follow up	N	X-ray	
Sayegh K[45]	2009	44	M	Diagnostic Radiology	Canada	L	V	Peri-cardiac floating rib	routine CT scan to follow up on a known left renal mass	N	X-ray, CT	along the left ventricular wall; first floating rib
Carvalho FHG[46]	2012	51	F	Radiology	Brasil	L	Ⅱ	Arising from 4th proximal rib	Chest pain	N	X-ray,CT+3D	Thoracic scoliosis
Basarslan F[47]	2012	6	F	Pediatrics	Turkey	R	Ⅰ A	anterior aspect of the T7 vertebral body and protruding into the thorax cavity	respiratory tract infection	N	X-ray, CT+3D	Scoliosis; rib protrudes into the thorax cavity ; lower lung collapse
Kayıran	2013	8	M	Pediatric	Turkey	R	Ⅰ A	3rd rib to the anterior portion of	runny nose and	Y	X-ray,	thick fibrous tissue

SM[48]								the sixth rib	coughing		CT	retracts the diaphragm
Mahajan PS[49]	2013	43	M	Radiology	Qatar	L	Ⅰ A +Ⅲ+ Ⅳ	Left 2nd Supernumerary rib and 3rd bifid Intrathoracic Ribs Arising from the 2nd and 3rd costovertebral articulations	high grade fever, cough, and chest pain	N	X-ray, CT+3D	T2-3 Bloc vertebra; hypo-plastic left lung; Fibrous attachment to the pleura
Prados J[50]	2013	57	M	Anatomy and Embryology	Spain	L	Ⅱ	4 ribs from most dorsal part of the normal ribs	autopsy	N		Only case with four intrathoracic rib
Coyan G[51]	2016	23	F	Thoracic and Cardiovascular	Turkey	L	Ⅰ B	the anterior surface of the T4; thin membrane attached to posterior chest wall	Moving chest pain and tightness; cough	N	CT+3D	**Relevant symptoms** Robotic assisted VATS
Padmanabhan A[52]	2016	10	M	Respiratory; Radiodiagnosis	India	L	Ⅰ A	adjacent to the T2-3 vertebrae	asthma	N	X-ray, CT+3D	
Abdollahifar MA[53]	2017	3	M	Anatomical Sciences and Biology	Iran	R	Ⅲ	the middle part of inferior border of the right 2nd rib inferior-interior toward 7th rib	CT scan for URTI	N	X-ray, CT+3D	
Ikeda M[54]	2017	19	F	Respiratory Medicine	Japan	L	Ⅰ A	abnormal rib articulated with the left 6th rib	routine chest radiograph	N	CT+3D	made a course within the lung parenchyma
Ahmadipour S[55]	2017	3	M	Pediatrics	Iran	R	Ⅱ	Arising from 2nd rib, down towards 6th rib	cold and cough	N	X-ray, CT+3D	
Kuyumcu G[56]	2017	-	M	Radiology	USA	R	Ⅱ	inferiomedial part of the 3rd rib	cough and chills	N	X-ray, CT+3D	
Kabakus	2017	19	F	Radiology	Turkey	L	Ⅱ	left 2nd rib head	dyspnea on exertion	N	CT+3D	

IM[57]									and fatigue			
	2017	59	F	Radiology	Turkey	R	Ⅱ	the right 5th rib head	cervical paraganglioma	N	CT+3D	expansion of the rib head
Thakare P[4]	2018	48	F	Pulmonary Medicine	India	R	Ⅱ	Posterior aspect of expanded right 4th rib	Dyspnoea, wheeze and cough with mucoid expectoration	Y	X-ray, CT+3D	linked two ribs from 4th to 6th rib
Xue XH[58]	2020	13	M	Orthopedics	China	B	ⅠB+ Ⅲ+ Ⅳ	Anterior aspect of T5 vertebra, toward on anterior inferolaterally	Pre-op routine check up for spine deformity	Y	X-ray, CT+3D	Bilateral bifid intrathoracic rib protruding into the thorax cavity; kyphoscoliosis
Muise ED	2020	17	F	Pulmonary Medicine	USA	L	Ⅱ	inferior aspect of the left third rib	cough, chest pain, and dyspnea	Y	X-ray, CT+3D	
Canan A	2020	43	F	Radiology	USA	R	Ⅱ	the proximal part and head of the right posterior fifth rib and extending inward and inferiorly along the posterior costal surface of the right hemithorax	endometrioid carcinoma of the uterus was evaluated for the presence of metastasis	N	X-ray, CT+3D	

Notes: F: female; M: male; R: right side; L: left side; B:bilateral; Y: yes; N: no; TB: tuberculosis; VATS: video-assisted thoracic surgery; SLE: systemic lupus erythematosus; ED: emergency department; THA: total hip arthroplasty; BFV: butterfly vertebra.
